# M3Drop: dropout-based feature selection for scRNASeq

**DOI:** 10.1093/bioinformatics/bty1044

**Published:** 2018-12-24

**Authors:** Tallulah S Andrews, Martin Hemberg

**Affiliations:** Department of Cellular Genetics, Wellcome Trust Sanger Institute, Hinxton, Cambridgshire, UK

## Abstract

**Motivation:**

Most genomes contain thousands of genes, but for most functional responses, only a subset of those genes are relevant. To facilitate many single-cell RNASeq (scRNASeq) analyses the set of genes is often reduced through feature selection, i.e. by removing genes only subject to technical noise.

**Results:**

We present M3Drop, an R package that implements popular existing feature selection methods and two novel methods which take advantage of the prevalence of zeros (dropouts) in scRNASeq data to identify features. We show these new methods outperform existing methods on simulated and real datasets.

**Availability and implementation:**

M3Drop is freely available on github as an R package and is compatible with other popular scRNASeq tools: https://github.com/tallulandrews/M3Drop.

**Supplementary information:**

Supplementary data are available at *Bioinformatics* online.

## 1 Introduction

Single-cell RNASeq (scRNASeq) has made it possible to analyze the transcriptome from individual cells. In a typical scRNASeq experiment for human or mouse, ∼10 000 genes will be detected. Most genes, however, are not relevant for understanding the underlying biological processes, and an important computational challenge is to select the most relevant features. Feature selection improves the signal to noise ratio and the computational efficiency of downstream analyses, such as clustering or pseudotime inference, by reducing the number of genes under consideration. However, unsupervised feature selection remains difficult due to the high technical variability and the low detection rates of scRNASeq experiments.

In this work, we introduce M3Drop a software package for performing feature selection for scRNASeq data. M3Drop implements several existing feature selection methods, including identifying highly variable genes (Brennecke *et al.*, 2013), GiniClust (Jiang *et al.*, 2016), PCA-based e.g. (Macosko *et al.*, 2015), and introduces two novel feature selection methods which identify genes with unusually high numbers of zeros, also called ‘dropouts’, among their observations. The advantage of using the dropout-rate over variance is that the former can be estimated more accurately due to much lower sampling noise (Supplementary Fig. S1).

These novel methods exploit the observation that dropout-rates per gene are strongly correlated with gene expression level (Pierson and Yau, 2015; Kharchenko *et al.*, 2014). Due to the non-linear nature of this relationship, averaging expression level and dropout rate across a heterogeneous cell population results in differentially expressed (DE) genes being shifted above the expected curve. Hence, biologically relevant features can be identified as outliers above the null expectation.

## 2 Materials and methods 

M3Drop implements two dropout-based feature selection methods with specific models for the null expectation, that are tailored to either read-counts from full-transcript sequencing protocols, such as Smartseq2 (Picelli *et al.*, 2013), or unique molecular identifier (UMI) counts from tag-based protocols, such as 10X Chromium (Zheng *et al.*, 2017). The first method fits a Michaelis-Menten function to the relationship between mean expression (*S*) and dropout-rate (P_dropout_) (**M3Drop**). Since the Michaelis-Menten function has a single parameter (K_M_), we can test the hypothesis that the gene-specific K_i_ is equal to the K_M_ that was fit for the whole transcriptome. This can be done by propagating errors on both observed dropout rate and observed mean expression to estimate the error of each K_i_. The significance can then be evaluated using a t-test (see: Supplementary Methods). We confirmed that the M3Drop model fits diverse Smartseq/2 scRNASeq datasets (Supplementary Fig. S3d–f).

The second method fits a library-size adjusted negative binomial model (see: Supplementary Methods) similar to those used previously to model variability in UMI-tagged data (Grün *et al.*, 2014) and bulk RNASeq data (Anders *et al.*, 2013). Genes with high dropout rates (**NBDrop**) or high dispersions (**NBDisp**) can be identified as features. Unlike M3Drop, NBDrop does not account for errors in the estimated mean expression levels. Thus, it is not as well suited for data with small-sample sizes and/or high amplification noise, as is typical of full-transcript, plate-based protocols (Islam *et al.*, 2014). We confirmed that the NBDrop model fits diverse tag-based scRNASeq datasets (Supplementary Fig. S3a–c). Since M3Drop integrates multiple feature selection methods into a single package, we are also able to calculate consensus features by averaging gene ranking across all six implemented feature selection methods.

## 3 Results

**Fig. 1.**
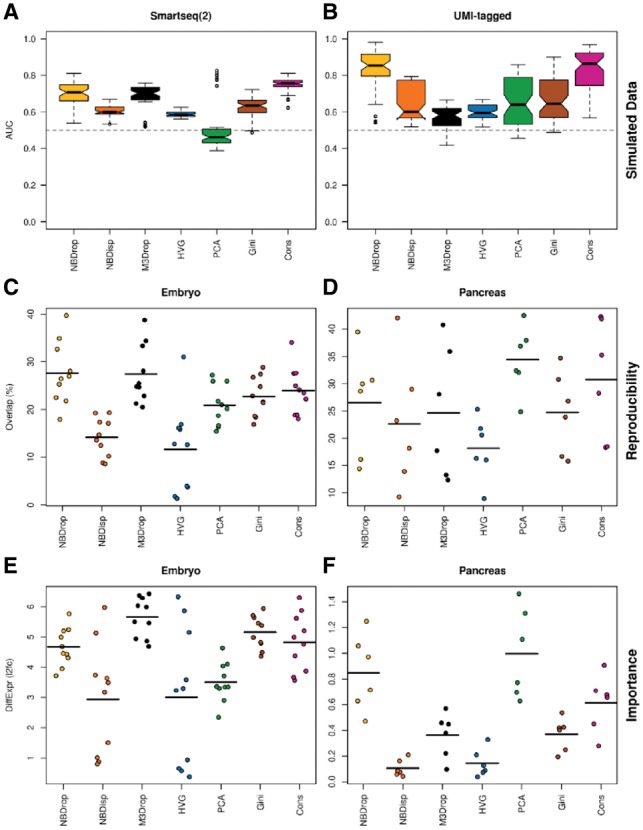
Comparison of feature selection methods. (**A** and **B**) Accuracy in identifying DE genes in simulated data. (**C** and **D**) Reproducibility of features across five mouse embryo and four human pancreas datasets. (**E** and **F**) Average fold-change in expression of reproducible features. (C–F) Each point represents a pair of datasets and the horizontal lines indicate the mean across all pairs. PCA scored genes by their loadings for the top components, Gini is the method used by GiniClust (Jiang *et al.*, 2016), Cons is the consensus across all other methods

We evaluated the performance of dropout-based feature selection compared to existing feature selection methods on data simulated from either a zero-inflated negative binomial (ZINB) fit to one of three different Smartseq/2 datasets (Fig. 1A) or data simulated from a negative binomial model with variability in library size (LS-NB) fit to one of three different UMI-tagged datasets (Fig. 1B). We generated a total of 108 simulated datasets and test each method’s ability to identify DE genes. For a fair comparison, we ranked genes by significance (if available) or effect-size and calculate the area under the ROC curve (AUC) from these rankings.

Both dropout-based feature selection methods, NBDrop and M3Drop, performed significantly better than variance-based feature selection on the ZINB simulations, as did the consensus features (Fig. 1A). Furthermore, NBDrop and consensus features significantly outperformed other feature selection methods on LS-NB simulations. Notably, the popular HVG method was only marginally better than random chance (AUC < 0.6). One potential disadvantage of dropout-based feature selection is that they may be unable to detect highly expressed genes since these may have no dropouts, even when they are DE across cell populations. However, when we binned data by expression level, we found that dropout-based feature selection performance only dropped below variance-based feature selection for the top 5% most highly expressed genes in our simulations (Supplementary Fig. S4). This corresponds to a mean expression level of >1000 reads/cell or >64 umis/cell, which is rare in the most datasets.

To demonstrate unsupervised feature selection in real datasets, we considered five datasets examining early mouse embryo development and four datasets examining human pancreas (Supplementary Table S1). Since these datasets are derived from the same biological system, we expect the most significant features to be reproducible (Fig. 1C and D). To ensure reproducibility was not due to technical biases, we also considered the magnitude of the log-fold-change in expression across the annotated cell-types for the selected features (Fig. 1E and F). Both dropout-based feature selection methods were more reproducible and identified genes with larger fold changes that other methods in the mouse embryo datasets (Fig. 1C and E). Reproducibility was highly dependent on the paired datasets for the pancreas data, since half are UMI-tagged data and half are full-transcript data. However, genes identified by NBDrop had larger fold changes than those of the other methods, except for PCA. This result is consistent with our previous findings that high-dropout genes are superior to high-variance genes for mapping across datasets (Kiselev *et al.*, 2018). The advantage of dropout-based holds for both discrete clustering and pseudotime analysis as it is the only method that preserves both distinct biological stages and the developmental trajectory of when combining developmental datasets (Supplementary Fig. S8).

## Funding

This work has been supported by the Wellcome Trust Sanger Core Funding and the Chan Zuckerberg Initiative DAF, Grant Reference 183501.

*Conflict of Interest*: none declared.

## Supplementary Material

bty1044_Supplementary_Data
