# Supplementary material for: M3Drop: dropout-based feature selection for scRNASeq
Source: Bioinformatics. 2018 Dec 24;35(16):2865–7. doi: 10.1093/bioinformatics/bty1044 (PMC6691329; doi:10.1093/bioinformatics/bty1044)
Supplement: bty1044_Supplementary_Data [file bty1044_supplementary_data.zip › bty1044-Suppl_data/Bioinformatics_Supplementary_Code.pdf]

Script: SetUpPanc.R

```
require("RColorBrewer")
require("gplots")
require("M3Drop")
require("matrixStats")
require("Matrix")
require("SingleCellExperiment")
require("scater")
N_fs = 2000;
MM_col = "black"
Depth_col = "goldenrod1"
NBVar_col = "#ff7f00"
pca_1_col = "#33a02c"
pca_2_col = "#b2df8a"
hvg_1_col = "#1f78b4"
hvg_2_col = "#a6cee3"
gini_col = "#b15928"
cor_col = "#6a3d9a"
cons_col = "violetred"

my_row_mean_aggregate <- function (mat, groups) {
    MAT <- as.matrix(mat)
    x <- split(seq(ncol(MAT)), groups)
    result <- sapply(x, function(a) rowMeans(MAT[, a]))
    return(result)
}

common_genes <- vector()
for (f in files) {
        obj <- readRDS(f)
        obj <- toSingleCellExperiment(obj)
        if (length(common_genes) == 0) {
                common_genes <- rownames(obj)
        } else {
                common_genes <- common_genes[common_genes %in% rownames(obj)]
        }
}

files <- c("baron-human.rds", "muraro_new.rds", "segerstolpe.rds", "xin.rds")

cell_types <- c("alpha", "beta", "delta", "gamma", "ductal", "acinar", "mesenchyme",
"mesen", "PSC", "activated_stellate", "quiescent_stellate")

# Plotting setup
method_names_all = c("NBDrop", "NBDisp", "M3Drop", "HVG", "PCA", "Cor", "Gini",
"Cons")
method_names = c("NBDrop", "NBDisp", "M3Drop", "HVG", "PCA", "Gini", "Cons")
```

```
exclude_methods <- function(features) {
        colnames(features) <- method_names_all[method_names_all != "All"];
        features <- features[, colnames(features) %in% method_names]
        return(features);
}
my_datasets = list()
i = 0;
for (f in files) {
        i <- i+1
        obj <- readRDS(f)
        obj <- toSingleCellExperiment(obj)
        obj <- obj[, as.character(obj$cell_type1) %in% cell_types]
        obj <- obj[rowSums(assays(obj)[["logcounts"]]) > 0,]
        obj <- obj[rownames(obj) %in% common_genes,] # Added 14 Sept 2018

        assays(obj)[["norm"]] <- 2^assays(obj)[["logcounts"]] -1
        if (!("counts" %in% names(assays(obj)))) {
                assays(obj)[["counts"]] <- NBumiConvertData(obj)
        }
        # Mean Expression each cell_type each dataset.
        # FS each dataset
        Features <- Consensus_FS(assays(obj)[["counts"]], assays(obj)[["norm"]],
include_cors=FALSE); # order: DANBdrop, DANBvar, M3Drop, Hvg, pca, cor, Gini, Cons
        Features <- exclude_methods(Features);
        expression <- my_row_mean_aggregate(assays(obj)[["logcounts"]],
factor(obj$cell_type1))
        my_datasets[[i]] <- list(FS=Features, expr=expression);
}

names(my_datasets) <- c("baron", "muraro", "seger", "xin")
saveRDS(my_datasets, "Panc_CrossDatasets.rds")
```

Script: DevoSetUp.R

```
library("M3Drop")

# Clean up datasets
biotype_ensg = read.table("mmusculus_80_biotypes.txt", header=F)
biotype_sym = read.table("mmusculus_80_biotypes_symbol.txt", header=F)
pseudo = c(as.character(biotype_ensg[grep("pseudogene",biotype_ensg[,2]),1]),
as.character(biotype_sym[grep("pseudogene",biotype_sym[,2]),1]))
rm(biotype_ensg)
rm(biotype_sym)
normalize_data <- function(data, labels=1:length(data[1,]), is.counts=TRUE) {
        # Genes : detected (non-zero) in at least 3 cells & not pseudo genes &
average normalized expr > 10^-5
        # Cells : have at least 2000 detected genes (non-zero)
        pseudo_genes = rownames(data) %in% pseudo;
```

```
        data = data[!pseudo_genes,];

        Ndetected = colSums(data > 0);
        data=data[,Ndetected>2000];
        labels=labels[Ndetected>2000];

        detected = rowSums(data > 0) > 3;
        data = data[detected,];

        spikes = grep("ercc",rownames(data), ignore.case=TRUE)
        if (is.counts) {
                if (length(spikes) > 0) {
                        totreads = colSums(data[-c(spikes),])
                } else {
                        totreads = colSums(data)
                }
                cpm = t(t(data)/totreads)*1000000;
                lowExpr = rowMeans(cpm) < 10^-5;
                cpm=cpm[!lowExpr,];
                return(list(data=cpm, labels=labels));
        }
        lowExpr = rowMeans(data) < 10^-5;
        data=data[!lowExpr,];
        return(list(data=data, labels=labels));
}
#Mapping read names
map = read.table("Mmus_Gene_Name_Mapping_Ensembl80.out", header=T)
ensg2symbol <- function(x) {
        new = as.character(map[match(x, map[,1]),2])
        new[is.na(new)] = as.character(x[is.na(new)])
        new[duplicated(new)] = x[duplicated(new)]
        return(new)
}


convert_to_integer <- function(mat) {
        mat <- ceiling(as.matrix(mat))
        storage.mode(mat) <- "integer"
        mat = mat[rowSums(mat) > 0,]
        return(mat)
}

get_ICM_TE_assignments<- function(x, labels) {
        data <- log(x+1)/log(2);
        # TE & ICM assignment
        TE_markers=c("Elf5","Eomes","Cdx2")
        ICM_markers = c("Sox2","Pou5f1","Nanog")
        scale <- function(x) {(x-mean(x))/sd(x)}
```

```
        ICMscore = rowMeans(apply(data[rownames(data) %in% ICM_markers,], 1, scale))
        TEscore = rowMeans(apply(data[rownames(data) %in% TE_markers,], 1, scale))
        blasts = (grepl("blast", labels) | grepl("32cel", labels))
        new_lab = as.character(labels);
        if (sum(blasts) > 0) {
                new_lab[blasts & ICMscore > TEscore] = "ICM"
                new_lab[blasts & ICMscore < TEscore] = "TE"
        }
        names(new_lab) <- colnames(x)
        return(new_lab);
}
# Deng
load("/lustre/scratch117/cellgen/team218/TA/scRNASeqDatasets/Deng_embryo_clean.RData"
)
counts_list = normalize_data(Deng_embyro_list$data, labels = Deng_embyro_list$labels,
is.counts = FALSE)
norm_list = normalize_data(Deng_embyro_list$data, labels = Deng_embyro_list$labels,
is.counts = TRUE)

DENG <- list(counts=counts_list$data, norm=norm_list$data, labels=counts_list$labels)
DENG$labels <- get_ICM_TE_assignments(DENG$norm, labels=DENG$labels)
rm(Deng_embyro_list); rm(counts_list); rm(norm_list);

# Zhong
zhong = read.table("GSE57249_fpkm_ZHONG.txt", header=TRUE);
zhong = zhong[!duplicated(zhong[,1]),]
rownames(zhong) = zhong[,1]
zhong = zhong[,2:length(zhong[1,])]
zhong = as.matrix(zhong);

zhong_labels = read.table("GSE57249_labels_ZHONG.txt");
zhong_labels <- as.character(unlist(zhong_labels))
ultralow = which(rowMeans(zhong) < 10^-5)
zhong = zhong[-ultralow,]
zhong_list = normalize_data(zhong, labels=zhong_labels, is.counts=FALSE)
zhong_count = convert_to_integer(zhong_list$data);
zhong_list$data <- zhong_list$data[rownames(zhong_list$data) %in%
rownames(zhong_count),]

BIASE <- list(counts=zhong_count, norm=zhong_list$data, labels=zhong_list$labels)
BIASE$labels <- get_ICM_TE_assignments(BIASE$norm, labels=BIASE$labels)
rm(zhong); rm(zhong_list); rm(zhong_count); rm(zhong_labels); rm(ultralow);
# Xue
Xue_data = read.table("GSE44183_mouse_expression_mat.txt", header=TRUE)
Xue_labels = read.table("GSE44183_mouse_expression_labels.txt", header=FALSE)
Xue_labels = as.character(unlist(Xue_labels))
Xue_labels[Xue_labels == "2cellmixed"] = "2cellmix"
Xue_labels[Xue_labels == "4cellmixed"] = "4cellmix"
```

```
Xue_labels[Xue_labels == "8cellmixed"] = "8cellmix"
Xue_labels[Xue_labels == "Pronucleus"] = "pronuc"
Xue_list = normalize_data(Xue_data, labels=Xue_labels, is.counts=FALSE)
Xue_count = convert_to_integer(Xue_list$data);
Xue_list$data <- Xue_list$data[rownames(Xue_list$data) %in% rownames(Xue_count),]
XUE <- list(counts=Xue_count, norm=Xue_list$data, labels=Xue_list$labels)
XUE$labels <- get_ICM_TE_assignments(XUE$norm, labels=XUE$labels)
rm(Xue_data); rm(Xue_list); rm(Xue_count); rm(Xue_labels)
# Fan
Fan_data = read.table("GSE53386_matrix_fpkms.tsv", header=TRUE)
Fan_labels = read.table("GSE53386_labels.tsv", header=FALSE)
Fan_labels = as.character(unlist(Fan_labels))
Fan_labels[Fan_labels == "a-AM_treated_2-cell"] = "AM_2cell"
Fan_labels[Fan_labels == "2-cell"] = "2cell"
Fan_labels[Fan_labels == "4-cell"] = "4cell"
Fan_labels[Fan_labels == "8-cell"] = "8cell"
Fan_list = normalize_data(Fan_data, labels=Fan_labels, is.counts=FALSE)
Fan_count = convert_to_integer(Fan_list$data)
Fan_list$data <- Fan_list$data[rownames(Fan_list$data) %in% rownames(Fan_count),]
FAN <- list(counts=Fan_count, norm=Fan_list$data, labels=Fan_list$labels)
FAN$labels <- get_ICM_TE_assignments(FAN$norm, labels=FAN$labels)
rm(Fan_data); rm(Fan_list); rm(Fan_count); rm(Fan_labels)

# Goolam
Goolam_data = read.table("Goolam_et_al_2015_count_table.tsv", header=T)
Goolam_labels = read.table("Goolam_et_al_2015_count_table_labels.tsv", header=F)
Goolam_list = normalize_data(Goolam_data, unlist(Goolam_labels[1,]), is.counts=TRUE)
Goolam_counts = normalize_data(Goolam_data, unlist(Goolam_labels[1,]),
is.counts=FALSE)
Goolam_list$labels = (as.character(Goolam_list$labels))
Goolam_list$labels[Goolam_list$labels =="32cell"] = "blast"
rownames(Goolam_counts$data) = ensg2symbol(rownames(Goolam_counts$data))
rownames(Goolam_list$data) = ensg2symbol(rownames(Goolam_list$data))

GOO <- list(counts=Goolam_counts$data, norm=Goolam_list$data,
labels=Goolam_list$labels)
GOO$labels <- get_ICM_TE_assignments(GOO$norm, labels=GOO$labels)
rm(Goolam_data); rm(Goolam_list); rm(Goolam_counts); rm(Goolam_labels)
# Make Consistent
common_genes <- rownames(DENG$norm)[
                rownames(DENG$norm) %in% rownames(BIASE$norm) &
                rownames(DENG$norm) %in% rownames(XUE$norm) &
                rownames(DENG$norm) %in% rownames(FAN$norm) &
                rownames(DENG$norm) %in% rownames(GOO$norm)]
common_genes = sort(common_genes);

Combined_counts <- cbind(
        DENG$counts[match(common_genes, rownames(DENG$counts)),],
```

```
        BIASE$counts[match(common_genes, rownames(BIASE$counts)),],
        FAN$counts[match(common_genes, rownames(FAN$counts)),],
        XUE$counts[match(common_genes, rownames(XUE$counts)),],
        GOO$counts[match(common_genes, rownames(GOO$counts)),]
        )
Combined_norm <- cbind(
        DENG$norm[match(common_genes, rownames(DENG$norm)),],
        BIASE$norm[match(common_genes, rownames(BIASE$norm)),],
        FAN$norm[match(common_genes, rownames(FAN$norm)),],
        XUE$norm[match(common_genes, rownames(XUE$norm)),],
        GOO$norm[match(common_genes, rownames(GOO$norm)),]
        )
dataset = c(
        rep("D", times=ncol(DENG$counts)),
        rep("B", times=ncol(BIASE$counts)),
        rep("F", times=ncol(FAN$counts)),
        rep("X", times=ncol(XUE$counts)),
        rep("G", times=ncol(GOO$counts))
        )
truth = c(
        as.character(unlist(DENG$labels)),
        as.character(unlist(BIASE$labels)),
        as.character(unlist(FAN$labels)),
        as.character(unlist(XUE$labels)),
        as.character(unlist(GOO$labels))
        )
truth[grep("ocyte",truth)] = "zygote"
truth[grep("nuc",truth)] = "zygote"
truth[grep("AM",truth)] = "zygote"
truth[grep("early2",truth)] = "zygote"
truth[grep("2cell",truth)] = "2cell"
truth[grep("4cell",truth)] = "4cell"
truth[grep("8cell",truth)] = "8cell"
truth[grep("orula",truth)] = "16cell"
truth[grep("TE",truth)] = "blast"
truth[grep("ICM",truth)] = "blast"

DEVO = list(counts=Combined_counts, norm=Combined_norm, labels=truth, batch=dataset)
rm(Combined_counts); rm(Combined_norm); rm(truth); rm(dataset); rm (common_genes);

require("RColorBrewer")
require("gplots")
require("M3Drop")
require("matrixStats")
require("Matrix")
MM_col = "black"
Depth_col = "goldenrod1"
NBVar_col = "#ff7f00"
```

```
pca_1_col = "#33a02c"
pca_2_col = "#b2df8a"
hvg_1_col = "#1f78b4"
hvg_2_col = "#a6cee3"
gini_col = "#b15928"
cor_col = "#6a3d9a"
cons_col = "violetred"

my_row_mean_aggregate <- function (mat, groups) {
    MAT <- as.matrix(mat)
    x <- split(seq(ncol(MAT)), groups)
    result <- sapply(x, function(a) rowMeans(MAT[, a]))
    return(result)
}


N_fs = 2000;

# Plotting setup
Stage = factor(DEVO$labels, levels=c("zygote", "2cell", "4cell", "8cell", "16cell",
"blast"))
Source = factor(DEVO$batch)
batch_pch = c(17,1,18,15,16)
stage_col = brewer.pal("Set2", n=length(levels(Stage)));
ICM_col="cornflowerblue"
TE_col="forestgreen"
method_names_all = c("NBDrop", "NBDisp", "M3Drop", "HVG", "PCA", "Cor", "Gini",
"Cons")
method_names = c("NBDrop", "NBDisp", "M3Drop", "HVG", "PCA", "Gini", "Cons")

exclude_methods <- function(features) {
        colnames(features) <- method_names_all;
        features <- features[, colnames(features) %in% method_names]
        return(features);
}

# Mean Expression each cell_type each dataset.
# FS each dataset
Features <- Consensus_FS(DENG$counts, DENG$norm, include_cors=FALSE); # order:
DANBdrop, DANBvar, M3Drop, Hvg, pca, cor, Gini, Cons
Features <- exclude_methods(Features);
expression <- my_row_mean_aggregate(log2(DENG$norm+1), DENG$labels)
X_FS <- Consensus_FS(XUE$counts, as.matrix(XUE$norm));
X_FS <- exclude_methods(X_FS);
XUE$labels <- sub("mix", "", XUE$labels)
X_expr <- my_row_mean_aggregate(log2(XUE$norm+1), factor(XUE$labels))
F_FS <- Consensus_FS(FAN$counts, as.matrix(FAN$norm), include_cors=FALSE);
F_FS <- exclude_methods(F_FS);
```

```
F_expr <- my_row_mean_aggregate(log2(FAN$norm+1), factor(FAN$labels))
B_FS <- Consensus_FS(BIASE$counts, as.matrix(BIASE$norm), include_cors=FALSE);
B_FS <- exclude_methods(B_FS);
B_expr <- my_row_mean_aggregate(log2(BIASE$norm+1), factor(BIASE$labels))
G_FS <- Consensus_FS(GOO$counts, as.matrix(GOO$norm), include_cors=FALSE);
G_FS <- exclude_methods(G_FS);
G_expr <- my_row_mean_aggregate(log2(GOO$norm+1), factor(GOO$labels))

my_datasets = list(Deng=Features, Biase=B_FS, Fan=F_FS, Xue=X_FS, Goolam=G_FS)
stuff=list(DENG=list(FS=Features, expr=expression), XUE=list(FS=X_FS, expr=X_expr),
FAN=list(FS=F_FS, expr=F_expr), BIASE=list(FS=B_FS, expr=B_expr), GOO=list(FS=G_FS,
expr=G_expr))
saveRDS(stuff, "Devo_CrossDatasets.rds")
```

Script: MainFigure.R

```
require("M3Drop")
require("matrixStats")
require("RColorBrewer")
require("gplots")
require("Matrix")

MM_col = "black"
Depth_col = "goldenrod1"
NBVar_col = "#ff7f00"
pca_1_col = "#33a02c"
pca_2_col = "#b2df8a"
hvg_1_col = "#1f78b4"
hvg_2_col = "#a6cee3"
gini_col = "#b15928"
cor_col = "#6a3d9a"
cons_col = "violetred"

# Set up
dir = "/lustre/scratch117/cellgen/team218/TA/Simulations_Temporary_Files/";
type = c("Umi","Full")
datasets = list(umi=c("blisch", "kirsch", "lin"),full=c("Ola","Buet","Pollen"))
seeds = c(1001, 1234, 6789)
subpop = c(1,10,20,30,40,50)
case_names = c("DE","DVar","HVar")
method_names_all = c("NBDrop", "NBDisp", "M3Drop", "HVG", "PCA", "Cor", "Gini",
"Cons")
method_names = c("NBDrop", "NBDisp", "M3Drop", "HVG", "PCA", "Gini", "Cons")
col_vec = c(Depth_col, NBVar_col, MM_col, hvg_1_col, pca_1_col, gini_col, cons_col);

UMI_base = paste("Umi_",rep(datasets$umi, each=3),"_", rep(seeds, times=3), sep="")
UMI_base = paste(rep(UMI_base, each=length(subpop)),rep(subpop,
times=length(UMI_base)), sep="_")
```

```
FULL_base = paste("Full_",rep(datasets$full, each=3),"_", rep(seeds, times=3),
sep="")
FULL_base = paste(rep(FULL_base, each=length(subpop)),rep(subpop,
times=length(FULL_base)), sep="_")

Umi_DE_files <- paste(UMI_base, "_DE_FS.rds", sep="")
Umi_DV_files <- paste(UMI_base, "_DVar_FS.rds", sep="")
Umi_HV_files <- paste(UMI_base, "_HVar_FS.rds", sep="")
Full_DE_files <- paste(FULL_base, "_DE_FS.rds", sep="")
Full_DV_files <- paste(FULL_base, "_DVar_FS.rds", sep="")
Full_HV_files <- paste(FULL_base, "_HVar_FS.rds", sep="")

exclude_methods <- function(features) {
        features <- features[, -c(6)]
        return(features);
}

vs_Exp_lvl <- function(Mega_Feature_table, method_names) {
        Exp_lvl <- Mega_Feature_table$Mean
        Feature_table <- Mega_Feature_table[,1:length(method_names)]

        my_breaks = c(min(Exp_lvl)-1, quantile(Exp_lvl, probs=seq(from=0, to=1,
by=0.05)))
        binned = cut(Exp_lvl, my_breaks, right=TRUE);

        auc_tab_all <- matrix(0, nrow=length(levels(binned)),
ncol=length(method_names));
        auc_tab_top <- matrix(0, nrow=length(levels(binned)),
ncol=length(method_names));
        xes <- vector(length=length(levels(binned)));

        for(i in 2:length(levels(binned))) {
                b = levels(binned)[i]
                xval = median(Exp_lvl[binned==b])
                AUCs = fast_total_AUCs(Mega_Feature_table[binned==b,], method_names);
                AUCs =AUCs[2,]
                auc_tab_all[i,] = AUCs
                xes[i] = xval
        }
        colnames(auc_tab_top) = method_names;
        colnames(auc_tab_all) = method_names;

        return(list(xes=xes, y_tab = auc_tab_all, y_tab_top=auc_tab_top))
}
fast_total_AUCs <- function(Mega_Feature_table, method_names) {
        Feature_table <- Mega_Feature_table[Mega_Feature_table$is.Pos==TRUE |
Mega_Feature_table$is.Neg,]
```

```
        Truth = Feature_table$is.Pos
        Feature_table <- Feature_table[,1:length(method_names)]
        reranked <- apply(Feature_table, 2, rank)
        # using Mann-Whitney U test
        N1 = sum(Truth);
        N2 = sum(!Truth);
        Rs = colSums(reranked[Truth,])
        Us = Rs - N1*(N1+1)/2
        # CIs
        AUCs = 1-Us/(N1*N2);
        Q1s = AUCs/(2-AUCs)
        Q2s = 2*AUCs^2/(1+AUCs)
        SEs = sqrt( (AUCs*(1-AUCs) + (N1-1)*(Q1s-AUCs^2) +
(N2-1)*(Q2s-AUCs^2))/(N1*N2) )
        CI_low = AUCs-1.96*SEs; CI_low[CI_low < 0] = 0;
        CI_high = AUCs+1.96*SEs; CI_high[CI_high > 1] = 1;
        Out_tab <- rbind(CI_low, AUCs, CI_high);
        colnames(Out_tab) = colnames(Feature_table)
        rownames(Out_tab) = c("CI_lo", "AUC", "CI_hi")
        return(Out_tab);
}
fast_partial_AUCs <- function(Mega_Feature_table, method_names, topN=2000) {
        Pos_Truth <- rownames(Mega_Feature_table)[Mega_Feature_table$is.Pos]
        Neg_Truth <- rownames(Mega_Feature_table)[Mega_Feature_table$is.Neg]
        Feature_table <- Mega_Feature_table[,1:length(method_names)]

        get_Feature_list <- function(FS_ranks, n=2000) {
                names(FS_ranks) <- rownames(Feature_table);
                FS_ranks <- FS_ranks[order(FS_ranks)]
                genes <- names(FS_ranks)[1:n]
                return(genes);
        }
        Feature_list <- apply(Feature_table, 2, get_Feature_list,
n=nrow(Feature_table))
        Feature_list <- Feature_list[1:topN,]

        fast_AUC <- function(Genes, Pos_Truth, Neg_Truth) {
                Genes <- Genes[Genes %in% Pos_Truth | Genes %in% Neg_Truth];
                truth <- Genes %in% Pos_Truth;

                R = 1:length(Genes);
                U = sum(R[truth])-sum(truth)*(sum(truth)+1)/2
                N1 = sum(truth)
                N2 = sum(!truth);
                if (N1 == 0) {return(c(0,0,0))}
                if (N2 == 0) {return(c(1,1,1))}
                AUC = 1-U/(N1*N2);
                Q1 = AUC/(2-AUC)
```

```
		Q2 = 2*AUC^2/(1+AUC)
		SE = sqrt((AUC*(1-AUC)+ (N1-1)*(Q1-AUC^2)+(N2-1)*(Q2-AUC^2))/(N1*N2))
		return(c(max(0, AUC-1.96*SE),AUC, min(1, AUC+1.96*SE)));
	}
	out = apply(Feature_list, 2, fast_AUC, Pos_Truth= Pos_Truth, 
Neg_Truth=Neg_Truth)
	rownames(out) = c("CI_low", "AUC", "CI_high")
	return(out)
}

summarize_files <- function(file_vec) {
	out_cor_tab <- matrix(0, ncol=length(method_names)+1, 
nrow=length(method_names)+1)
	out_cor_Pos_tab <- matrix(0, ncol=length(method_names)+1, 
nrow=length(method_names)+1)
	exp_lvl <-list(xes=0, y_tab=rep(0, times=length(method_names)))
	tot_AUCs <- vector(length=length(method_names))
	top_AUCs <- vector(length=length(method_names))

	for (f in file_vec) {
		Features <- readRDS(file=paste(dir, f, sep=""));
		Features <- exclude_methods(Features);

		expout <- vs_Exp_lvl(Features, method_names)
		exp_lvl$xes = c(exp_lvl$xes, expout$xes)
		exp_lvl$y_tab = rbind(exp_lvl$y_tab, expout$y_tab)

		tot_AUCs = rbind(tot_AUCs, fast_total_AUCs(Features, 
method_names)[2,])
		top_AUCs = rbind(top_AUCs, fast_partial_AUCs(Features, 
method_names)[2,])
	}
	return(list(AllCor = c(), PosCor = c(), vsExpr=exp_lvl, AllAUC=tot_AUCs, 
TopAUC=top_AUCs))
}

VsExpressionPlot <- function(OUT) {
	plot(1,1, col="white", xlim=log(c(min(OUT$vsExpr$xes[OUT$vsExpr$xes>0]), 
		max(OUT$vsExpr$xes)))/log(2), ylim=c(0,1), 
		xlab="Log2 Mean Expression", ylab="AUC")

	for (ver_j in c(1,2)) {
		for (meth_i in 1:length(col_vec)) {
			tmp_x = OUT$vsExpr$xes
			tmp_y = OUT$vsExpr$y_tab[,meth_i]
			tmp_y <- tmp_y[tmp_x>0]
			tmp_x <- tmp_x[tmp_x>0]
			tmp_x <- log(tmp_x)/log(2)
```

```
                        my_col = col2rgb(col_vec[meth_i])
                        my_col = rgb(my_col[1,1], my_col[2,1], my_col[3,1], alpha=5, 
maxColorValue=255)
                        if (ver_j == 1) {
                                points(tmp_x, tmp_y, col=my_col, pch=16)
                        } else {
                                smspline = smooth.spline(tmp_x, tmp_y, spar=0.9)
                                lines(smspline, col=col_vec[meth_i], lwd=3)
                        }
                }
        }
        score <- colMeans(OUT$vsExpr$y_tab)
        reorder = order(-score)
        abline(h=0.5, lty=2, lwd=1, col="black")
        legend("bottomleft", paste(method_names[reorder], " (", round(score[reorder], 
digits=2), ")", sep=""), col=col_vec[reorder], lty=1, lwd=3, bty="n", ncol=2)
}

VsSubPopPlot <- function(OUT, subpop, all=TRUE) {
        if (all) {
                AUC_tab <- OUT$AllAUC
                ylabel="AUC"
        } else {
                AUC_tab <- OUT$TopAUC
                ylabel="pAUC (n=2000)"
        }
        AUC_tab <- AUC_tab[-1,]
        subpop_lab = 1:nrow(AUC_tab) %% length(subpop)
        subpop_lab[subpop_lab==0] <- length(subpop)
        require("CellTypeProfiles")
        average <- t(my_row_mean_aggregate(t(AUC_tab), subpop_lab))
        matplot(subpop, average, col=col_vec, type="l", ylim=c(0,1), xlab="Population 
size (%)", ylab=ylabel, lty=1, lwd=3)
        abline(h=0.5, lty=2, col="black", lwd=1)

        score <- colMeans(average)
        reorder = order(-score)
        legend("bottomleft", paste(method_names[reorder], " (", round(score[reorder], 
digits=2), ")", sep=""), col=col_vec[reorder], lty=1, lwd=3, bty="n", ncol=2)
}

OUTFullDE <- summarize_files(Full_DE_files);
OUTUmiDE <- summarize_files(Umi_DE_files);

# Application Note

png("OneFigurePaper_Sim_DE_vs_Expr.png", width=5*2, height=5, units="in", res=350)
par(mfrow=c(1,2))
```

```
VsExpressionPlot(OUTFullDE)
title(main="ZINB")
mtext("A", side=2, at=1, font=2, las=2, cex=1.2, line=3)

VsExpressionPlot(OUTUmiDE)
title(main="LS-NB")
mtext("B", side=2, at=1, font=2, las=2, cex=1.2, line=3)
dev.off()

png("OneFigurePaper_Sim_DE_vs_Subpop.png", width=5*2, height=5*2, units="in",
res=350)
par(mfrow=c(2,2))
VsSubPopPlot(OUTFullDE, subpop, all=FALSE)
title(main="ZINB")
mtext("A", side=2, at=1, font=2, las=2, cex=1.2, line=3)

VsSubPopPlot(OUTUmiDE, subpop, all=FALSE)
title(main="LS-NB")
mtext("B", side=2, at=1, font=2, las=2, cex=1.2, line=3)

VsSubPopPlot(OUTFullDE, subpop, all=TRUE)
title(main="ZINB")
mtext("C", side=2, at=1, font=2, las=2, cex=1.2, line=3)

VsSubPopPlot(OUTUmiDE, subpop, all=TRUE)
title(main="LS-NB")
mtext("D", side=2, at=1, font=2, las=2, cex=1.2, line=3)
dev.off()

### All together AUC plots ###

OUTFullDV <- summarize_files(Full_DV_files);
OUTUmiDV <- summarize_files(Umi_DV_files);

OUTFullHV <- summarize_files(Full_HV_files);
OUTUmiHV <- summarize_files(Umi_HV_files);

my_make_boxplot_data <- function(AUC_table, type="DE") {
        require("reshape2")
        box_data_DE <- AUC_table[-1,]
        box_data_DE <- melt(box_data_DE);
        box_data_DE[,1] = rep(type, times=nrow(box_data_DE));
        colnames(box_data_DE) = c("Type", "Method", "AUC")
        return(box_data_DE)
}

All_Full <- my_make_boxplot_data(OUTFullDE$AllAUC)
All_UMI <- my_make_boxplot_data(OUTUmiDE$AllAUC)
```

```
Top_Full <- my_make_boxplot_data(OUTFullDE$TopAUC)
Top_UMI <- my_make_boxplot_data(OUTUmiDE$TopAUC)

# One Figure Panels A & B
par(mfrow=c(3,2))
par(mar=c(5,4,3,1))
boxplot(AUC ~ Method, data=All_Full, col=c(col_vec, col_vec, col_vec), ylab="AUC",
ylim=c(0,1), notch=TRUE, las=2) # All -> Top for top 200
abline(h=0.5, lty=2, col="grey35")
title(main="Smartseq(2)")

par(mar=c(5,2,3,3))
boxplot(AUC ~ Method, data=All_UMI, col=c(col_vec, col_vec, col_vec), ylab="AUC",
ylim=c(0,1), notch=TRUE, las=2) # All -> Top for top 200
abline(h=0.5, lty=2, col="grey35")
title(main="UMI-tagged")
mtext("Simulated Data", side=4, line=1, at=0.5, font=2)

N_fs = 2000;
method_names = c("NBDrop", "NBDisp", "M3Drop", "HVG", "PCA", "Gini", "Cons")

# Plotting setup
devo <- readRDS("Devo_CrossDatasets.rds")
panc <- readRDS("Panc_CrossDatasets2.rds")

# Devo
l2fc_tab <- list()
for (i in 1:length(devo)) {
        low <- apply(devo[[i]]$expr, 1, min)
        hi <- apply(devo[[i]]$expr, 1, max)
        l2fc <- hi-low
        l2fc_tab[[i]] <- l2fc;
}
names(l2fc_tab) <- names(devo);

pairs <- combn(names(devo), 2)
meth <- colnames(devo[[1]]$FS)
out_devo_pairs <- matrix(-1, nrow=length(colnames(devo[[1]]$FS)), ncol=ncol(pairs));
out_devo_l2fc <- matrix(-1, nrow=length(colnames(devo[[1]]$FS)), ncol=ncol(pairs));
for (j in 1:ncol(pairs)) {
        for (i in 1:length(meth)) {
                m <- meth[i]
                fs1 <- rownames(devo[[pairs[1,j]]]$FS)[devo[[pairs[1,j]]]$FS[,m] <=
N_fs]
                fs2 <- rownames(devo[[pairs[2,j]]]$FS)[devo[[pairs[2,j]]]$FS[,m] <=
N_fs]
                out_devo_pairs[i, j] <- sum(fs1 %in% fs2)/N_fs
```

```
                consistent <- fs1[fs1 %in% fs2]
                de1 <- l2fc_tab[[pairs[1,j]]]
                de2 <- l2fc_tab[[pairs[2,j]]]
                out_devo_l2fc[i, j]<-(median(de1[names(de1) %in% consistent])
                         + median(de2[names(de2) %in% consistent])) / 2
        }
}
rownames(out_devo_pairs) <- method_names
rownames(out_devo_l2fc) <- method_names

# Pancreas
l2fc_tab <- list()
for (i in 1:length(panc)) {
        low <- apply(panc[[i]]$expr, 1, min)
        hi <- apply(panc[[i]]$expr, 1, max)
        l2fc <- hi-low
        l2fc_tab[[i]] <- l2fc;
}
names(l2fc_tab) <- names(panc);

pairs <- combn(names(panc), 2)
meth <- colnames(panc[[1]]$FS)
out_panc_pairs <- matrix(-1, nrow=length(colnames(panc[[1]]$FS)), ncol=ncol(pairs));
out_panc_l2fc <- matrix(-1, nrow=length(colnames(panc[[1]]$FS)), ncol=ncol(pairs));
for (j in 1:ncol(pairs)) {
        for (i in 1:length(meth)) {
                m <- meth[i]
                fs1 <- rownames(panc[[pairs[1,j]]]$FS)[panc[[pairs[1,j]]]$FS[,m] <=
N_fs]
                fs2 <- rownames(panc[[pairs[2,j]]]$FS)[panc[[pairs[2,j]]]$FS[,m] <=
N_fs]
                out_panc_pairs[i, j] <- sum(fs1 %in% fs2)/N_fs

                consistent <- fs1[fs1 %in% fs2]
                de1 <- l2fc_tab[[pairs[1,j]]]
                de2 <- l2fc_tab[[pairs[2,j]]]
                out_panc_l2fc[i, j]<-(median(de1[names(de1) %in% consistent])
                         + median(de2[names(de2) %in% consistent])) / 2
        }
}
rownames(out_panc_pairs) <- method_names
rownames(out_panc_l2fc) <- method_names

method_names = c("NBDrop", "NBDisp", "M3Drop", "HVG", "PCA", "Gini", "Cons")
col_vec = c(Depth_col, NBVar_col, MM_col, hvg_1_col, pca_1_col, gini_col, cons_col);
xes_devo <- rep(1:nrow(out_devo_l2fc), time=ncol(out_devo_l2fc))
xes_panc <- rep(1:nrow(out_panc_l2fc), time=ncol(out_panc_l2fc))
```

```
my_jitter_plot <- function(table) {
        xes <- 1:nrow(table)
        plot(jitter(rep(xes, times=ncol(table))), as.vector(table),xaxt="n", ylab="",
bg=col_vec, pch=21, xlab="")
        mids <- rowMeans(table)
        arrows(xes-0.4, mids, xes+0.4, mids, len=0, lwd=2)
        axis(1, at=xes, labels=rownames(table), las=2);
}

png("One_Figure_Paper_mainfigv2.png", width=4*2, height=3.5*3, units="in", res=350)
par(mfrow=c(3,2))
par(mar=c(5,4,3,1))
boxplot(AUC ~ Method, data=All_Full, col=c(col_vec, col_vec, col_vec), ylab="AUC",
ylim=c(0,1), notch=TRUE, las=2) # All -> Top for top 200
abline(h=0.5, lty=2, col="grey35")
title(main="Smartseq(2)")
mtext("A", side=3, line=1, at=0, font=2)

par(mar=c(5,2,3,3))
boxplot(AUC ~ Method, data=All_UMI, col=c(col_vec, col_vec, col_vec), ylab="AUC",
ylim=c(0,1), notch=TRUE, las=2) # All -> Top for top 200
abline(h=0.5, lty=2, col="grey35")
title(main="UMI-tagged")
mtext("Simulated Data", side=4, line=1, at=0.5, font=2)
mtext("B", side=3, line=1, at=0, font=2)

par(mar=c(5,4,3,1))
my_jitter_plot(out_devo_pairs*100)
title(main="Embryo", ylab="Overlap (%)")
mtext("C", side=3, line=1, at=0.5, font=2)
par(mar=c(5,2,3,3))
my_jitter_plot(out_panc_pairs*100)
title(main="Pancreas", ylab="Overlap (%)")
mtext("D", side=3, line=1, at=0.5, font=2)
mtext("Reproducibility", side=4, line=1, at=25, font=2)

par(mar=c(5,4,3,1))
my_jitter_plot(out_devo_l2fc)
title(main="Embryo", ylab="DiffExpr (l2fc)")
mtext("E", side=3, line=1, at=0.5, font=2)
par(mar=c(5,2,3,3))
my_jitter_plot(out_panc_l2fc)
title(main="Pancreas", ylab="DiffExpr (l2fc)")
mtext("F", side=3, line=1, at=0.5, font=2)
mtext("Importance", side=4, line=1, at=0.75, font=2)
dev.off()
```

Script: DevoCombinedPCAs.R

```
require("RColorBrewer")
require("gplots")
require("M3Drop")
source("Colour_Scheme.R")
source("~/NetworkInferencePipeline/Dropouts/My_R_packages/M3D/R/Other_FS_functions.R"
)
require("matrixStats")
require("Matrix")
source("Load_Devo.R")
source("/nfs/users/nfs_t/ta6/NetworkInferencePipeline/Dropouts/My_R_packages/M3D/R/NB
_UMI.R")

N_fs = 2000;

# Plotting setup
Stage = factor(DEVO$labels, levels=c("zygote", "2cell", "4cell", "8cell", "16cell",
"blast"))
Source = factor(DEVO$batch)
batch_pch = c(17,1,18,15,16)
stage_col = brewer.pal("Set2", n=length(levels(Stage)));
ICM_col="cornflowerblue"
TE_col="forestgreen"
method_names_all = c("NBDrop", "NBDisp", "M3Drop", "HVG", "PCA", "Cor", "Gini",
"Cons", "All")
method_names = c("NBDrop", "NBDisp", "M3Drop", "HVG", "PCA", "Gini", "Cons", "All")

exclude_methods <- function(features) {
        colnames(features) <- method_names_all[method_names_all != "All"];
        features <- features[, colnames(features) %in% method_names]
        return(features);
}

# TE & ICM
clust_fxn <- function(x){hclust(x, method="ward.D2")}
dist_fxn <- dist;
K = 2;

Features <- Consensus_FS(DENG$counts, DENG$norm, include_cors=FALSE); # order:
DANBdrop, DANBvar, M3Drop, Hvg, pca, cor, Gini, Cons
Features <- exclude_methods(Features);
set.seed(1);
DENG$time <- as.character(DENG$labels)
DENG$time[grep("midblast", names(DENG$time))] <- "midblast"
DENG$time[grep("earlyblast", names(DENG$time))] <- "earlyblast"
DENG$time[grep("lateblast", names(DENG$time))] <- "lateblast"
```

```
#### Combined Features ####
method_names_all = c("NBDrop", "NBDisp", "M3Drop", "HVG", "PCA", "Cor", "Gini",
"Cons")
method_names = c("NBDrop", "NBDisp", "M3Drop", "HVG", "PCA", "Gini", "Cons")
X_FS <- Consensus_FS(XUE$counts, as.matrix(XUE$norm), include_cors=FALSE); # order:
DANBdrop, DANBvar, M3Drop, Hvg, pca, cor, Gini
X_FS <- exclude_methods(X_FS);
F_FS <- Consensus_FS(FAN$counts, as.matrix(FAN$norm), include_cors=FALSE);
F_FS <- exclude_methods(F_FS);
B_FS <- Consensus_FS(BIASE$counts, as.matrix(BIASE$norm), include_cors=FALSE);
B_FS <- exclude_methods(B_FS);
G_FS <- Consensus_FS(GOO$counts, as.matrix(GOO$norm), include_cors=FALSE);
G_FS <- exclude_methods(G_FS);

my_datasets = list(Deng=Features, Biase=B_FS, Fan=F_FS, Xue=X_FS, Goolam=G_FS)
n_meth = ncol(Features)
n_set = length(my_datasets)
my_Matrix = matrix(0, ncol=n_meth*n_set, nrow=nrow(DEVO$norm))
rownames(my_Matrix) = rownames(DEVO$norm);
common_genes = rownames(DEVO$norm);

my_names = vector(length=n_meth*n_set)
for(d in 1:n_set) {
        for(m in 1:n_meth) {
                my_features <- my_datasets[[d]]
                my_features <- my_features[rownames(my_features) %in% common_genes,]
                my_features <- rownames(my_features)[order(my_features[,m])]
                my_features <- my_features[1:N_fs];

                my_Matrix[,(d-1)*n_meth+m] = common_genes %in% my_features;
                name <- paste(names(my_datasets)[d],method_names[m], sep="-")
                my_names[(d-1)*n_meth+m] = name
        }
}
colnames(my_Matrix) = my_names

m3d = seq(from=3, to=n_meth*n_set, by=n_meth)
hvg = seq(from=4, to=n_meth*n_set, by=n_meth)
nb = seq(from=1, to=n_meth*n_set, by=n_meth)
nbv = seq(from=2, to=n_meth*n_set, by=n_meth)
gini = seq(from=6, to=n_meth*n_set, by=n_meth)
pc23 = seq(from=5, to=n_meth*n_set, by=n_meth)
cons = seq(from=8, to=n_meth*n_set, by=n_meth)

#### PCAs ####
png("SupplmentaryPCAs.png", width=8*3/4, height=8.5*3/4, units="in", res=300)
make_PCA <- function(gene_list) {
```

```
        toplot <- log(DEVO$norm[rownames(DEVO$norm) %in% gene_list,]+1)/log(2);
        PCA = prcomp(toplot);
        plot(PCA$rotation[,1], PCA$rotation[,2], col=stage_col[Stage],
pch=batch_pch[Source], xlab="", ylab="")
        title(xlab=paste("PC1 (",round(PCA$sdev[1]/sum(PCA$sdev)*100, digits=2),"
%)",sep=""), line=2)
        title(ylab=paste("PC2 (",round(PCA$sdev[2]/sum(PCA$sdev)*100, digits=2),"
%)",sep=""), line=2)
        dists <- dist(t(toplot), method="euclidean")
        htree <- hclust(dists, method="ward.D2")
        return(PCA);
}

par(mfrow=c(3,3))
method_names = c("NBDrop", "NBDisp", "M3Drop", "HVG", "PCA", "Gini", "Cons", "All")
col_vec = c(Depth_col, NBVar_col, MM_col, hvg_1_col, pca_1_col, gini_col, cons_col,
"grey50");
col_sets <- list(nb, nbv, m3d, hvg, pc23, gini, cons)
for (i in 1:n_meth) {
        par(mar=c(3,3,2,1));
        thing = make_PCA(rownames(my_Matrix[rowSums(my_Matrix[,col_sets[[i]]]) >
2,]));
        title(main=method_names[i])
}
par(mar=c(3,3,2,1));
all = make_PCA(rownames(my_Matrix))
title(main="All")
blank_plot <- function() {
        tmp <-  par("mar")
        par(mar=c(0,0,0,0))
        plot(1,1, col="white", xlim=c(0,1), ylim=c(0,1), xaxt="n", yaxt="n", main="",
xlab="", ylab="", bty="n")
        return(tmp);
}
blank_plot()
legend("topleft", fill=stage_col, levels(Stage), bty="n", title="Stage")
legend("bottomleft", pch=batch_pch, c("Biase", "Deng", "Fan", "Goo", "Xue"), bty="n",
title="Dataset")
dev.off()
```
