# Supplementary material for: M3Drop: dropout-based feature selection for scRNASeq
Source: Bioinformatics. 2018 Dec 24;35(16):2865–7. doi: 10.1093/bioinformatics/bty1044 (PMC6691329; doi:10.1093/bioinformatics/bty1044)
Supplement: bty1044_Supplementary_Data [file bty1044_supplementary_data.zip › bty1044-Suppl_data/Supplementary_Data_ApplicationNote.pdf]

Supplementary Methods

Dropout-based feature selection methods:

M3Drop

The expression of each gene was averaged across all cells including those with zero reads for a particular gene (S). Dropout rate was calculated as the proportion of cells with zero reads for that gene ($P_{dropout}$). We fit the Michaelis-Menten equation (Michaelis and Menten, 1913) to the relationship between these two variables

$$P_{dropout} = 1 - \frac{S}{K_M + S}$$

using maximum likelihood estimation as implemented by the mle2 function in the bbmle R package to obtain the global K_M across all genes. This model fits full-transcript data very well (Fig. S2 D-F). The Michaelis-Menten equation can be rearranged with K on the left hand side. This is useful for estimating a gene-specific K_j as

$$K_j = \frac{P_j * S_j}{1 - P_j}$$

Since K_j is a function of both the dropout and the mean expression, the measurement error for each K_j estimate was calculated using error propagation rules to combine errors on observed S and P:

$$\sigma_{K_j} = K_j * \sqrt{\left(\frac{\sigma_S}{S}\right)^2 + \left(\frac{\sigma_P}{P}\right)^2}$$

Where σ_S is the sample standard deviation of S and σ_P is the sample standard deviation of P. The K_j's were observed to be log-normally distributed around the globally fit K_M (Fig. S5). Thus, we tested each one against the global K_M that was fit to the entire dataset using a one-sided Z-test:

$$Z = \frac{log(K_j) - log(K_M)}{\sqrt{\sigma^2_{log(K_j)} + \sigma^2_{log(K_M)}}}$$

$\sigma^2_{log(K_M)}$ was estimated as the standard error of the residuals and added to $\sigma^2_{log(K_j)}$

$$\sigma_{\log(K_M)} = \frac{sd(\log(K_j) - \log(K_M))}{\sqrt{N}}$$

$$\sigma_{\log(K_j)} = \log(K_j) - \log(K_j - \sigma_{Kj})$$

The resulting p-values can be used to ascertain the significance of each feature or to rank genes in order of decreasing significance (increasing p-value). Since all computations are based on gene-level statistics the method scales linearly with the number of cells and number of genes.

NBDrop & NBDisp

Negative binomial models have been shown to fit normalized molecule counts from single-cell RNASeq data employing unique molecular identifiers, referred to as UMI-tagged data (Grün et al., 2014; Islam et al., 2014) . We modified the single negative binomial distribution to explicitly model the tagging/sequencing efficiency for each cell (l_j) as the relative total molecules observed in cell *j*.

$$l_j = \sum_i \hat{y}_{ij} / \sum_i \sum_j \hat{y}_{ij}$$

where $\hat{y}_{ij}$ is the number of UMIs observed for gene *i* in cell *j*. Thus, each observation is modelled as a negative binomial model with mean and variance equal to:

$$E(Y_{ij}) = \mu_{ij}$$

$$V(Y_{ij}) = \mu_{ij} + \frac{\mu_{ij}^2}{r_i}$$

The mean (μ_{ij}) and the gene-specific dispersion parameter (r_i) are estimated as:

$$\hat{\mu}_{ij} = \sum_j \hat{y}_{ij} * l_j$$

$$\hat{r}_j = \frac{t_i^2 \sum_j l_j^2}{(n_c - 1) \sum_j (\hat{y_{ij}} - \hat{\mu_{ij}})^2 - t_i}$$

where t_i is the total molecules counts for gene *i*, and n_c is the total number of cells. Genes with Poissonian behavior, which results in negative dispersion, were assigned a maximum dispersion of 10^{10}. To identify high dispersion genes using this model (**NBDisp**) we fit a linear regression between the log observed mean expression and the log estimated dispersion parameter across all genes with mean expression > 16 and non-Poissonian dispersions. The residuals from this regression was used to rank genes.

$$log(\hat{r}_j) = \beta_0 + \beta_1 * log(\hat{\mu}_{ij})$$

The probability of any given observation being a zero (dropout) is calculated as:

$$P(Y_{ij} = 0) = (1 + \hat{\mu}_{ij} / \hat{r}_j)^{-\hat{r}_j}$$

And the expected total dropouts per gene is:

$$E(D_i) = \sum_j P(Y_{ij} = 0)$$

To identify high dropout genes we substituted the expected dispersion calculated from β_0 and β_1 in the linear regression equation above for the gene-specific dispersions, and model the number of dropouts per gene as a binomial distribution. The p-value of the observed dropouts can be used to test significance of features or to rank them. This method also scales linearly in number of genes and cells.

Other Feature Selection Methods

The highly variable gene (**HVG**) method was proposed by Brennecke et al. (2013) (Brennecke et al., 2013). HVG fits a quadratic model to the relationship between mean expression and the coefficient of variation squared (CV^2) and outliers above the fitted curve are selected as features. Originally, the model was fit using ERCC spike-ins, but this can be problematic since many datasets do not contain spike-ins or may have technical issues which affect the consistency of spike-ins added to all experimental batches (Svensson et al., 2017). Thus for consistency we fit the model using all genes, based on the assumption that most genes exhibit only technical noise.

In addition, we considered feature selection based on the Gini Index (Gini, 1912) a common measure of skewness. We use the method proposed in GiniClust (Jiang et al., 2016) to account for the relationship between the Gini Index and the maximum observed expression and estimate a p-value for each gene (**Gini**).

As an alternative to the above methods which all rely on identifying outlier genes based summary statistics, we considered principal component analysis (**PCA**), which ranked genes by their loadings for the top PCs calculate for the whole dataset. Since the first PC is often highly correlated with technical noise (Hicks et al. 2015) we by used the loadings of PC2 and PC3 for real datasets and we used PC1 and PC2 for simulated datasets. Gene-gene correlations can also be used for feature selection. However this method is extremely slow with greater than quadratic scaling, whereas all other methods scale approximately linearly for sparse matrices (Baglama and Reichel 2005), thus we did not consider it further.

The consensus features (**Cons**) were calculated by ranking every gene according to the p.value or effect-size, if p.values were not calculated by the feature selection method (i.e. PCA and NBDisp). These ranks were then averaged across all feature selection methods, and that consensus score was used to rank the genes.

Single-cell RNASeq datasets

We considered 15 public scRNASeq datasets (**Table S1**). These were chosen to reflect a range of different dataset sizes, sequencing methods and cell-types. Datasets where the expression matrix consisted of raw read counts (or UMI counts) were normalized using counts per million except for NBDrop and

NBDisp. Quality control was performed prior to all analyses as follows. First, all genes annotated as processed pseudogenes in Ensembl (version 80) were removed and cells with fewer than 2000 detected genes were removed. Genes detected in fewer than 4 cells or with average normalized expression < 10^{-5} were excluded from consideration. For the Deng data, only single mouse embryo cells analyzed using the SmartSeq protocol were considered to avoid technical artefacts.

Simulated datasets

We simulated UMI-tagged data using the depth-adjusted negative binomial model fit to one of the three UMI-tagged datasets, Tung (Tung et al., 2017), Zeisel (Zeisel et al., 2015) and Klein (Klein et al., 2015). Mean gene expression levels were drawn from a log-normal distribution fit to the respective dataset. Cell-specific sequencing efficiency was drawn from a gamma distribution. Finally, gene-specific dispersions were calculated from the mean expression level using the power-law relationship fit to the respective dataset. Each simulated dataset contained 25,000 genes and 500 cells and were consistent with the original data (Fig. S6,S7). The code for generating these simulations is included in the M3Drop R package as the `NBumiSimulationTrifecta` function for the depth-adjusted negative binomial model.

We simulated full-transcript data using a zero-inflated negative binomial model fit to each of three full-transcript datasets, Pollen (Pollen et al., 2014), Buettner (Buettner et al., 2015), or Kolodziejczyk (Kolodziejczyk et al., 2015). As before, mean gene expression levels were drawn from a log-normal distribution and gene-specific dispersions were calculated from the mean expression level using a power-law relationship. Simulated expression values were inflated with zeros according mean expression using the Michaelis-Menten equation fit to the respective dataset. Since full-transcript data is generally obtained from fewer cells than UMI-tagged data, each simulated full-transcript dataset contained 200 cells and 25,000 genes and were consistent with the original data (Fig. S6,S7). The code for generating these simulations is included in the M3Drop R package as the `M3DropSimulationTrifecta` function for the zero-inflated negative binomial model.

Differentially expressed (DE) genes were added by increasing/decreasing the mean expression of each gene in a subset of the cells by a log base-2 fold change drawn from a normal distribution with mean = 0 and sd = 2. Dispersions were adjusted in the differentially expressing cells according to the fitted power-law relationship. We considered subpopulations containing 1%, 10%, 20%, 30%, 40% or 50% of the cells and three replicates for each of the six dataset and each subpopulation size were generated.

Genes with a greater than 5-fold increase or decrease in mean expression were considered ground truth DE genes respectively. Genes with less than absolute 1-fold change in mean or dispersion were considered unchanged.

Reproducibility of Features

We considered five full-transcript single-cell RNASeq datasets examining mouse embryonic development from fertilization to blastocyst and four datasets examining human pancreas (**Table S1**). Only genes detected in all five embryo datasets were retained, leaving 11,315 genes in total, or in all four pancreas

datasets, leaving 13,993 genes. The top 2000 ranked genes for each feature selection method were calculated and the overlap between each pair of datasets was determined. The magnitude of the difference of expression for the consistent genes across each pair of datasets was calculate as the average of the median log2 fold-change of the consistent genes in each dataset. Code for the analysis of these real datasets is included in Supplementary File 2.

Author Contributions
TA and MH conceived of the project and wrote the manuscript. TA developed the method, produced the code, analyzed and interpreted the data. MH supervised the work.

Acknowledgements
The authors would like to thank: Vladimir Kiselev, Davis McCarthy, Simon Andrews, and Tomislav Ilicic for their comments and suggestions for improving this manuscript.

Competing Financial Interests
The authors declare they have no competing interests.

Supplementary Tables

Table S1: Publicly available single-cell RNASeq datasets, normalization method and proportion of zero valued entries in the filtered expression matrix.

Dataset	Cell-types	Labels	Protocol	N	% Zero	Source
Tung	Human iPSCs	Donor individual	5' Seq UMIs -> (CPM)	768	49%	(Tung et al., 2017) GSE77288
Klein	Mouse ESC	Differentiatio n timepoint	CEL-Seq UMIs -> (CPM)	2448	63%	(Klein et al., 2015) GSE65525
Zeisel	Mouse brain	BackSPIN clustering	5' Seq UMIs -> (CPM)	2542	77%	(Zeisel et al., 2015) GSE60361
Pollen	Human cell lines & tissues	Cell line identity	Smartseq FPKMs	301	60%	(Pollen et al., 2014) SRP041736**
Buettner	Mouse ESC	Cell-cycle stage	Smartseq Counts -> (CPM)	279	51%	(Buettner et al., 2015) E-MTAB-2805
Kolodziej czk (Kolo)	Mouse ESCs	Growth media	Smartseq Counts -> (CPM)	406	51%	(Kolodziejczyk et al., 2015) E-MTAB-2600
Deng	Mouse embryos	Development timepoint	Smartseq Counts -> (CPM)	255	50%	(Deng et al., 2014) GSE45719
Biase	Mouse embryos	Development stage	Smartseq FPKMs	56	38%	(Biase et al., 2014) GSE57249
Fan	Mouse embryos	Development stage	SUPeR-seq FPKMs	69	46%	(Fan et al., 2015) GSE53386
Xue	Mouse embryos	Development stage	Poly-A selected RPKMs	17	30%	(Xue et al., 2013) GSE44183
Goolam	Mouse embryos	Development stage	SmartSeq2 Counts -> (CPM)	124	43%	(Goolam et al., 2016) E-MTAB-3321
Baron	Human pancreas	Clustering + cell-types markers	3' droplet-based UMIs-> (CPM)	8569	91%	(Baron et al., 2016) GSE84133

Muraro	Human pancreas	Clustering + cell-types markers	CEL-Seq2 UMIs->(CPM)	2122	73%	(Muraro et al., 2016) GSE85241
Segerstolpe	Human pancreas	Clustering + cell-types markers	Smartseq2 Counts->(CPM)	3514	82%	(Segerstolpe et al., 2016) E-MTAB-5061
Xin	Human pancreas	Clustering + cell-types markers	Fluidigm C1 FPKMs	1600	86%	(Xin et al., 2016) GSE81608

**UMI = Unique Molecular Identifier; FPKM = fragments per kilobase per million; CPM = count per million*

*** Processed data was provided by the authors*

Supplementary Figures

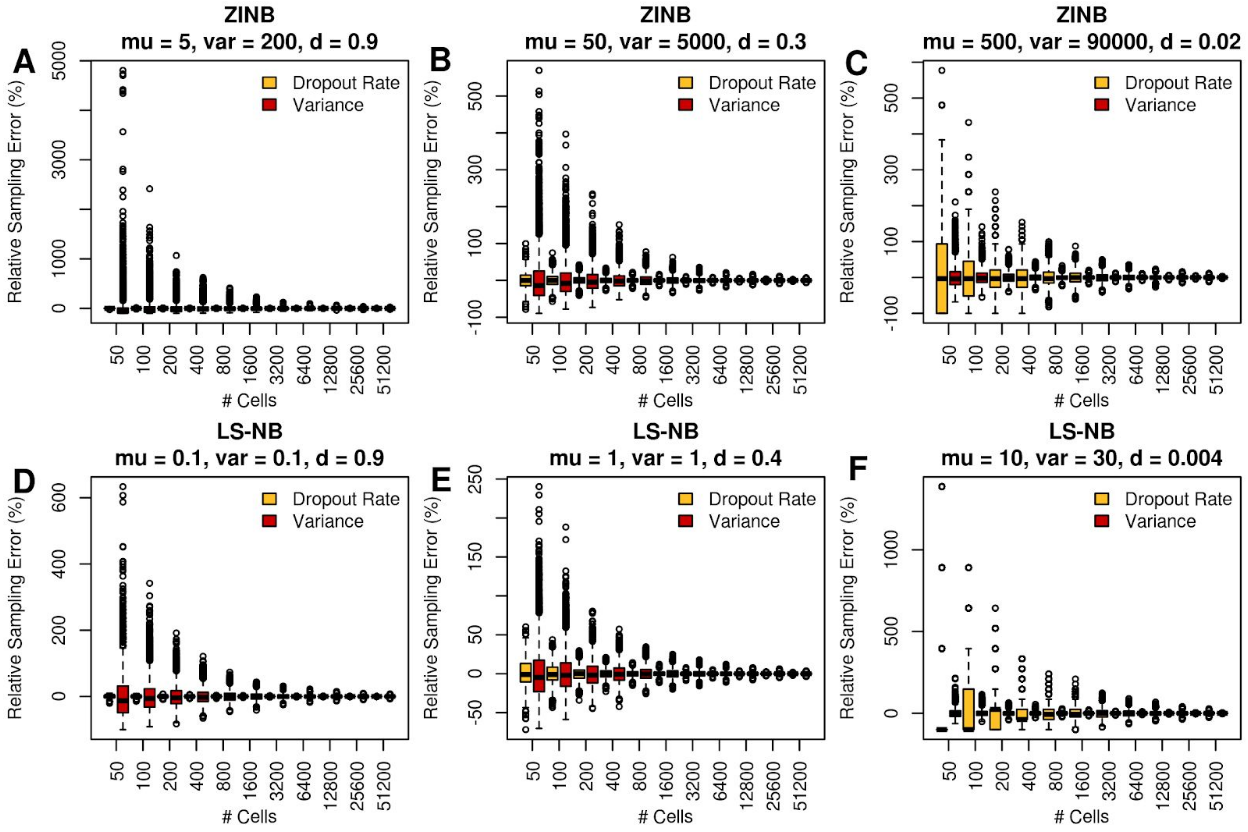

Fig. S1 Sampling noise for sample variance and sample dropout rate in a homogeneous population. Simulated expression values for 1,000,000 cells were downsampled without replacement 10,000 times. The percent error of the observed variance/dropout rate of the

downsampled values relative to the variance/dropout rate calculated over all 1,000,000 values was calculated. (**A-C**) Simulating from a zero-inflated negative binomial fit to Smartseq2 data. (**D-F**) Simulating from the library-size adjusted negative binomial fit to UMI-tagged data. mu = mean, var = variance, d = dropout-rate for all 1 million simulated cells.

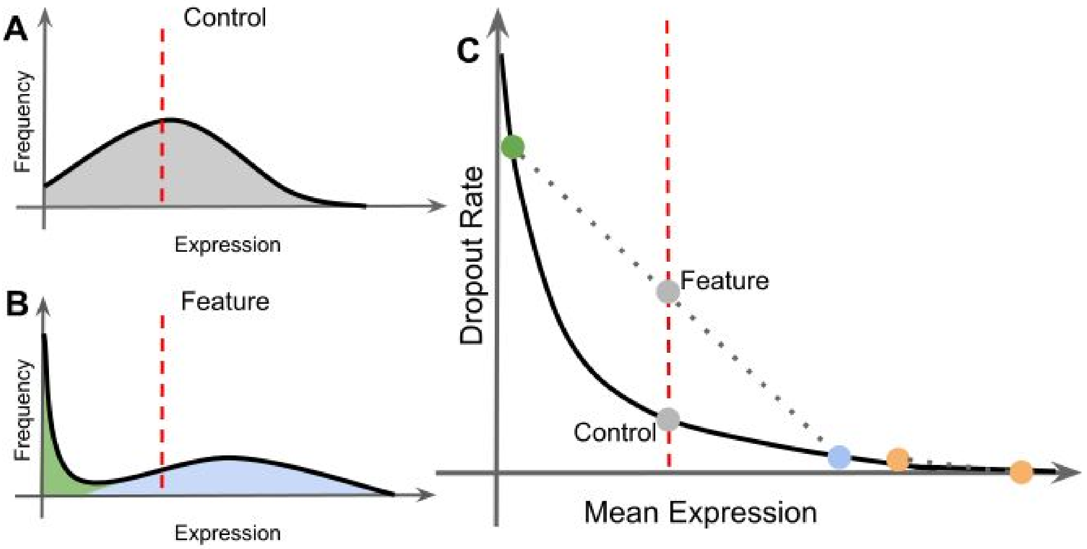

Fig. S2: Differentially expressed genes exhibit bimodal expression which increases the dropout rate relative to the mean expression. (**A & B**) Genes with the same mean expression (dashed red line), but (A) is expressed evenly across cells, whereas (B) is highly expressed in some cells (blue) and lowly expressed in others (green). (**C**)This leads to a surplus of dropouts since mean and dropout rate average linearly (dotted line) whereas the expectation (black line) is non-linear. Orange points indicate a gene with very high expression where differential expression leads to only a small increase in dropout-rate.

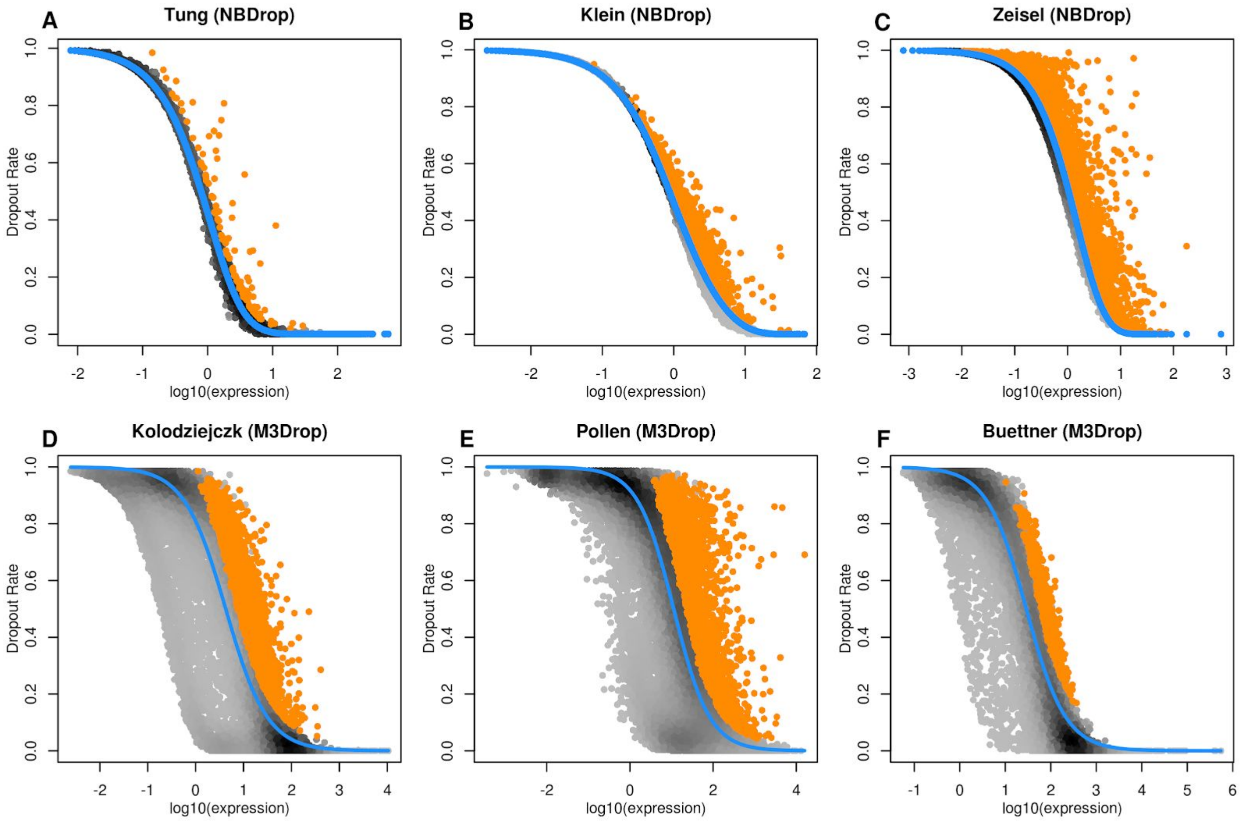

Fig. S3 Fitting NBDrop (**A-C**) and M3Drop (**D-F**) to three UMI-tagged and full-transcript scRNASeq datasets respectively. Each point is a gene coloured by the local density of points around it (black = high density). Blue line indicates the fitted relationship between mean and dropout rate from NBDrop or M3Drop respectively. Orange points are significant features using each method.

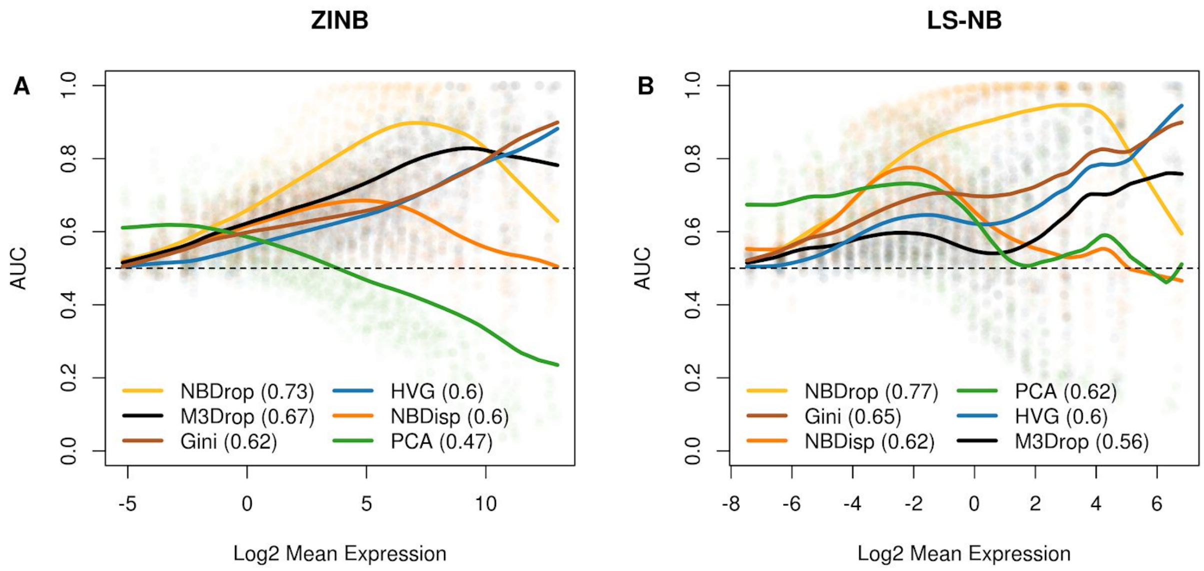

Fig. S4 Performance as measured by the AUC for all genes binned into 20 quantiles. Count matrices were simulated using a zero-inflated negative binomial (ZINB) or library-size adjusted negative binomial (LS-NB) fit to each of three full transcript and three umi-tagged datasets respectively. Three replicates of 25,000 genes each were performed for each datasets at each subpopulation size. Points are results for each bin from each replicate, lines are spline-smoothed trends for each feature selection method.

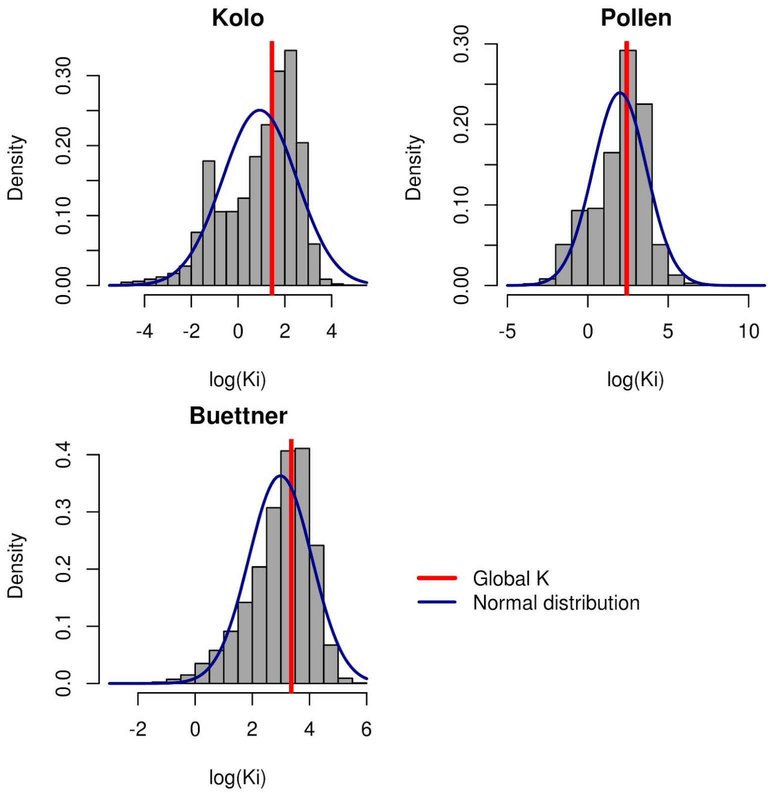

Fig. S5 Normal distribution of K_i around K_M

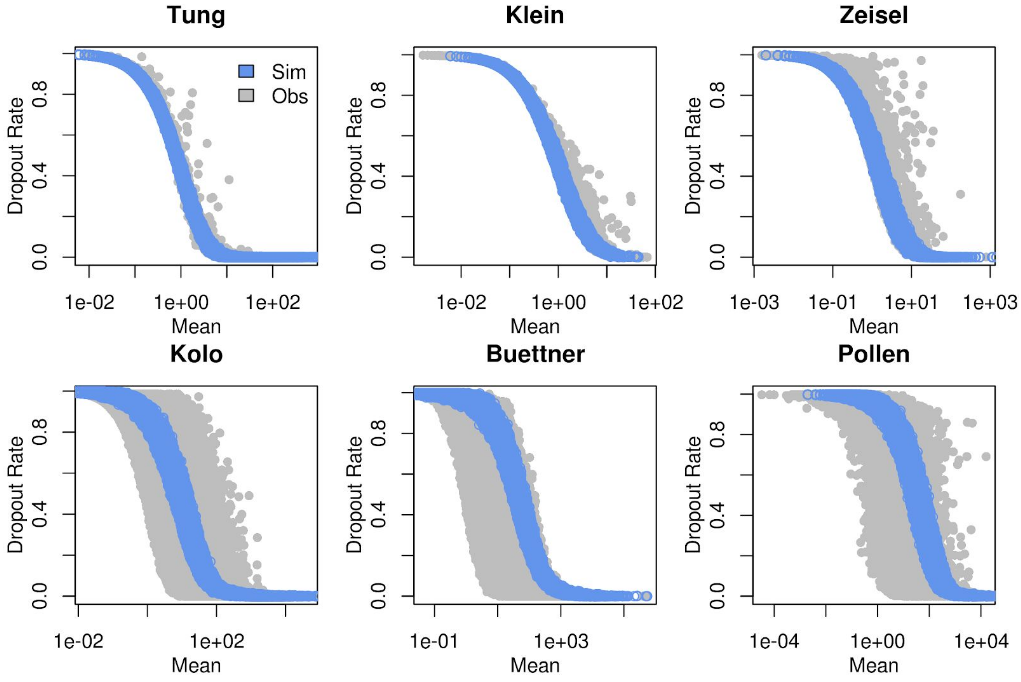

Fig. S6 Simulations recapitulate observed relationship between gene expression and dropout rate. Only genes with log fold changes smaller than one (ground truth negatives) are plotted for the simulated data (blue).

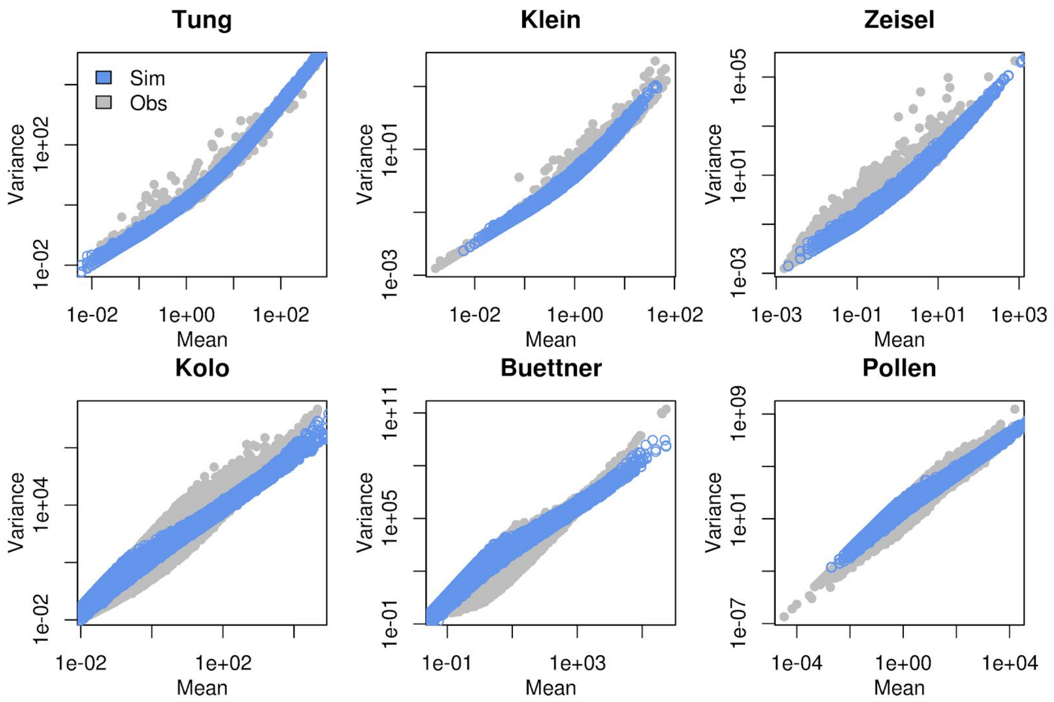

Fig. S7 Simulations recapitulate observed relationship between gene expression and variance. Only genes with log fold changes smaller than one (ground truth negatives) are plotted for the simulated data (blue).

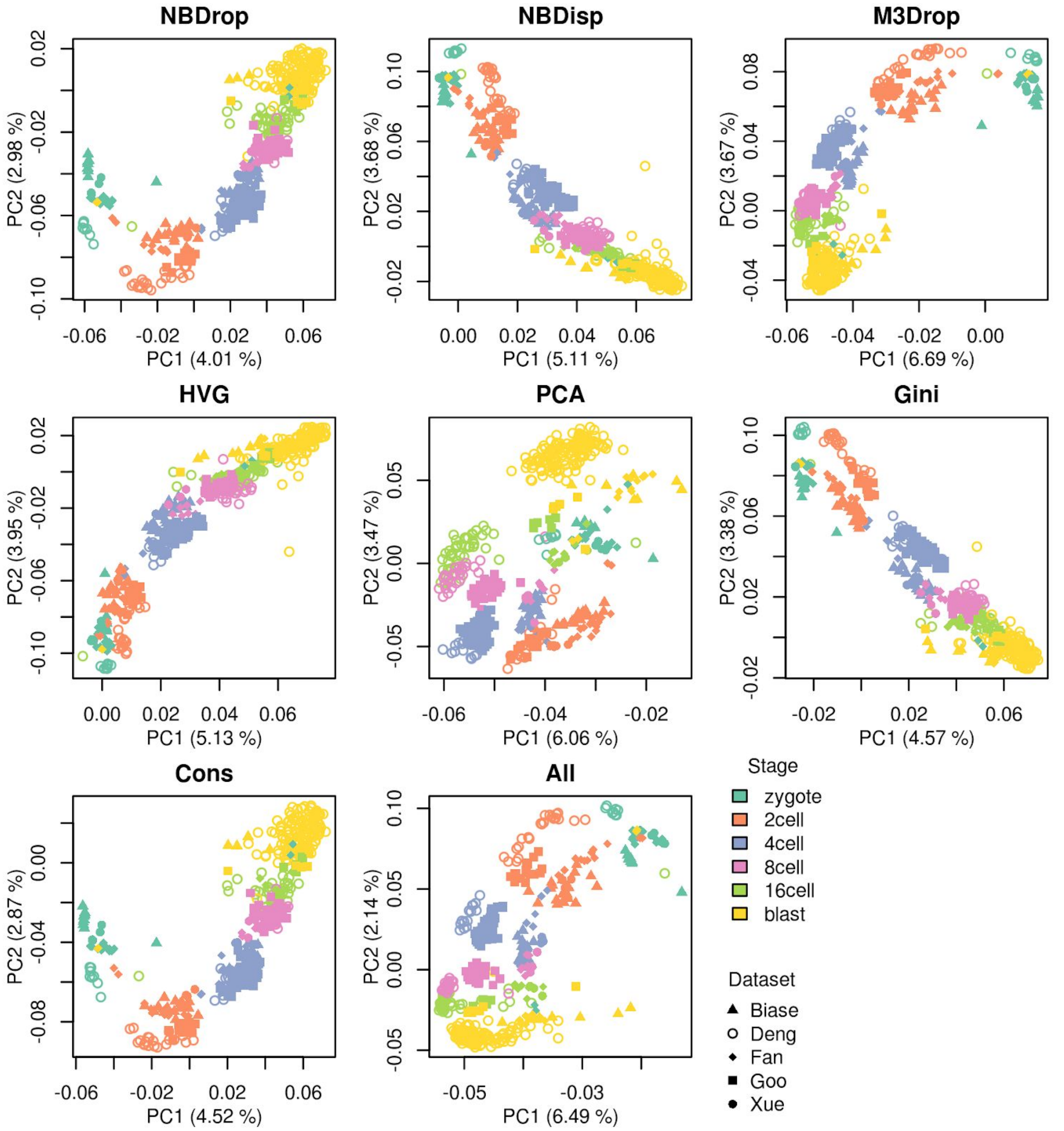

Fig. S8 Principal component analysis of the combined five mouse embryonic datasets using reproducibly identified features by each method. Genes were included if they were among the top 2000 features by a particular feature selection method in at least three of the datasets. Only Dropout-based features (NBDrop, M3Drop) preserved both the developmental trajectory and distinct developmental stages. In particular, PCA-based features did not preserve either biology signal. Variance-based methods (NBDisp, HVG, Gini) did not preserve the distinct stages, while not using any feature selection (All) resulted in batch effects dominating the first principal component.

References

Baron, M., Veres, A., Wolock, S.L., Faust, A.L., Gaujoux, R., Vetere, A., Ryu, J.H., Wagner, B.K., Shen-Orr, S.S., Klein, A.M., et al. (2016). A Single-Cell Transcriptomic Map of the Human and Mouse Pancreas Reveals Inter- and Intra-cell Population Structure. Cell Syst. *3*, 346–360.e4.

Biase, F.H., Cao, X., and Zhong, S. (2014). Cell fate inclination within 2-cell and 4-cell mouse embryos revealed by single-cell RNA sequencing. Genome Res. *24,* 1787–1796.

Brennecke, P., Anders, S., Kim, J.K., Kołodziejczyk, A.A., Zhang, X., Proserpio, V., Baying, B., Benes, V., Teichmann, S.A., Marioni, J.C., et al. (2013). Accounting for technical noise in single-cell RNA-seq experiments. Nat. Methods *10*, 1093–1095.

Buettner, F., Natarajan, K.N., Casale, F.P., Proserpio, V., Scialdone, A., Theis, F.J., Teichmann, S.A., Marioni, J.C., and Stegle, O. (2015). Computational analysis of cell-to-cell heterogeneity in single-cell RNA-sequencing data reveals hidden subpopulations of cells. Nat. Biotechnol. *33*, 155–160.

Deng, Q., Ramsköld, D., Reinius, B., and Sandberg, R. (2014). Single-cell RNA-seq reveals dynamic, random monoallelic gene expression in mammalian cells. Science *343*, 193–196.

Fan, X., Zhang, X., Wu, X., Guo, H., Hu, Y., Tang, F., and Huang, Y. (2015). Single-cell RNA-seq transcriptome analysis of linear and circular RNAs in mouse preimplantation embryos. Genome Biol. *16*, 148.

Gini, C. (1912). Variabilità e mutabilità. Reprinted in Memorie Di Metodologica Statistica (Ed. ….

Goolam, M., Scialdone, A., Graham, S.J.L., Macaulay, I.C., Jedrusik, A., Hupalowska, A., Voet, T., Marioni, J.C., and Zernicka-Goetz, M. (2016). Heterogeneity in Oct4 and Sox2 Targets Biases Cell Fate in 4-Cell Mouse Embryos. Cell *165*, 61–74.

Grün, D., Kester, L., and van Oudenaarden, A. (2014). Validation of noise models for single-cell transcriptomics. Nat. Methods *11*, 637–640.

Islam, S., Zeisel, A., Joost, S., La Manno, G., Zajac, P., Kasper, M., Lönnerberg, P., and Linnarsson, S. (2014). Quantitative single-cell RNA-seq with unique molecular identifiers. Nat. Methods *11*, 163–166.

Jiang, L., Chen, H., Pinello, L., and Yuan, G.-C. (2016). GiniClust: detecting rare cell types from single-cell gene expression data with Gini index. Genome Biol. *17*, 144.

Klein, A.M., Mazutis, L., Akartuna, I., Tallapragada, N., Veres, A., Li, V., Peshkin, L., Weitz, D.A., and Kirschner, M.W. (2015). Droplet barcoding for single-cell transcriptomics applied to embryonic stem cells. Cell *161*, 1187–1201.

Kolodziejczyk, A.A., Kim, J.K., Tsang, J.C.H., Ilicic, T., Henriksson, J., Natarajan, K.N., Tuck, A.C., Gao, X., Bühler, M., Liu, P., et al. (2015). Single Cell RNA-Sequencing of

Pluripotent States Unlocks Modular Transcriptional Variation. Cell Stem Cell *17*, 471–485.

Michaelis, L., and Menten, M.L. (1913). Die Kinetik der Invertinwirkung. Biochem. Z. *49*, 333–369.

Muraro, M.J., Dharmadhikari, G., Grün, D., Groen, N., Dielen, T., Jansen, E., van Gurp, L., Engelse, M.A., Carlotti, F., de Koning, E.J.P., et al. (2016). A Single-Cell Transcriptome Atlas of the Human Pancreas. Cell Syst. *3*, 385–394.e3.

Pollen, A.A., Nowakowski, T.J., Shuga, J., Wang, X., Leyrat, A.A., Lui, J.H., Li, N., Szpankowski, L., Fowler, B., Chen, P., et al. (2014). Low-coverage single-cell mRNA sequencing reveals cellular heterogeneity and activated signaling pathways in developing cerebral cortex. Nat. Biotechnol. *32*, 1053–1058.

Rand, W.M. (1971). Objective criteria for the evaluation of clustering methods. J. Am. Stat. Assoc. *66*, 846.

Segerstolpe, Å., Palasantza, A., Eliasson, P., Andersson, E.-M., Andréasson, A.-C., Sun, X., Picelli, S., Sabirsh, A., Clausen, M., Bjursell, M.K., et al. (2016). Single-Cell Transcriptome Profiling of Human Pancreatic Islets in Health and Type 2 Diabetes. Cell Metab. *24*, 593–607.

Svensson, V., Natarajan, K.N., Ly, L.-H., Miragaia, R.J., Labalette, C., Macaulay, I.C., Cvejic, A., and Teichmann, S.A. (2017). Power analysis of single-cell RNA-sequencing experiments. Nat. Methods *14*, 381–387.

Tung, P.-Y., Blischak, J.D., Hsiao, C.J., Knowles, D.A., Burnett, J.E., Pritchard, J.K., and Gilad, Y. (2017). Batch effects and the effective design of single-cell gene expression studies. Sci. Rep. *7*, 39921.

Ward, J.H. (1963). Hierarchical Grouping to Optimize an Objective Function. J. Am. Stat. Assoc. *58*, 236–244.

Xin, Y., Kim, J., Okamoto, H., Ni, M., Wei, Y., Adler, C., Murphy, A.J., Yancopoulos, G.D., Lin, C., and Gromada, J. (2016). RNA sequencing of single human islet cells reveals type 2 diabetes genes. Cell Metab. *24*, 608–615.

Xue, Z., Huang, K., Cai, C., Cai, L., Jiang, C., Feng, Y., Liu, Z., Zeng, Q., Cheng, L., Sun, Y.E., et al. (2013). Genetic programs in human and mouse early embryos revealed by single-cell RNA sequencing. Nature *500*, 593–597.

Zeisel, A., Muñoz-Manchado, A.B., Codeluppi, S., Lönnerberg, P., La Manno, G., Juréus, A., Marques, S., Munguba, H., He, L., Betsholtz, C., et al. (2015). Brain structure. Cell types in the mouse cortex and hippocampus revealed by single-cell RNA-seq. Science *347*, 1138–1142.
